# 5-Cyclo­hexyl-3-(4-fluoro­phenyl­sulfin­yl)-2-methyl-1-benzofuran

**DOI:** 10.1107/S1600536811007112

**Published:** 2011-03-02

**Authors:** Hong Dae Choi, Pil Ja Seo, Byeng Wha Son, Uk Lee

**Affiliations:** a Department of Chemistry, Dongeui University, San 24 Kaya-dong Busanjin-gu, Busan 614-714, Republic of Korea; b Department of Chemistry, Pukyong National University, 599-1 Daeyeon 3-dong, Nam-gu, Busan 608-737, Republic of Korea

## Abstract

In the title compound, C_21_H_21_FO_2_S, the cyclo­hexyl ring adopts a chair conformation. The 4-fluoro­phenyl ring makes a dihedral angle of 83.55 (4)° with the mean plane of the benzofuran fragment. In the crystal, mol­ecules are linked through weak inter­molecular C—H⋯O and C—H⋯π inter­actions. The crystal structure also exhibits aromatic π–π inter­actions between the furan rings of neighbouring mol­ecules [centroid–centroid distance = 3.541 (2) Å].

## Related literature

For the pharmacological activity of benzofuran compounds, see: Aslam *et al.* (2006 ▶); Galal *et al.* (2009 ▶); Khan *et al.* (2005 ▶). For natural products with benzofuran rings, see: Akgul & Anil (2003 ▶); Soekamto *et al.* (2003 ▶). For structural studies of related 3-(4-fluoro­phenyl­sulfin­yl)-2-methyl-1-benzofuran derivatives, see: Choi *et al.* (2010**a* ▶,*b* ▶,c* ▶).
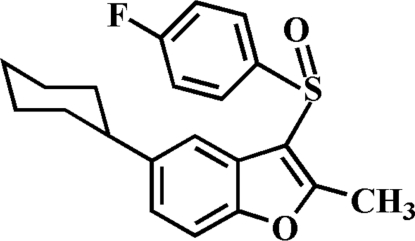


## Experimental

### 

#### Crystal data

C_21_H_21_FO_2_S
*M* *_r_* = 356.44
Triclinic, 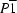

*a* = 9.2531 (2) Å
*b* = 10.1934 (2) Å
*c* = 10.8151 (2) Å
α = 81.127 (1)°
β = 66.716 (1)°
γ = 72.248 (1)°
*V* = 891.73 (3) Å^3^
*Z* = 2
Mo *K*α radiation
μ = 0.20 mm^−1^
*T* = 173 K
0.29 × 0.23 × 0.18 mm

#### Data collection

Bruker SMART APEXII CCD diffractometer
Absorption correction: multi-scan (*SADABS*; Bruker, 2009 ▶) *T*_min_ = 0.943, *T*_max_ = 0.964
15790 measured reflections
4073 independent reflections
3436 reflections with *I* > 2σ(*I*)
*R*_int_ = 0.027

#### Refinement

*R*[*F* ^2^ > 2σ(*F* ^2^)] = 0.040
*wR*(*F* ^2^) = 0.111
*S* = 1.04
4073 reflections
227 parameters
H-atom parameters constrained
Δρ_max_ = 0.60 e Å^−3^
Δρ_min_ = −0.27 e Å^−3^

### 

Data collection: *APEX2* (Bruker, 2009 ▶); cell refinement: *SAINT* (Bruker, 2009 ▶); data reduction: *SAINT*; program(s) used to solve structure: *SHELXS97* (Sheldrick, 2008 ▶); program(s) used to refine structure: *SHELXL97* (Sheldrick, 2008 ▶); molecular graphics: *ORTEP-3* (Farrugia, 1997 ▶) and *DIAMOND* (Brandenburg, 1998 ▶); software used to prepare material for publication: *SHELXL97*.

## Supplementary Material

Crystal structure: contains datablocks global, I. DOI: 10.1107/S1600536811007112/zq2090sup1.cif

Structure factors: contains datablocks I. DOI: 10.1107/S1600536811007112/zq2090Isup2.hkl

Additional supplementary materials:  crystallographic information; 3D view; checkCIF report

## Figures and Tables

**Table 1:** Hydrogen-bond geometry (Å, °) *Cg*1 is the centroid of the C2–C7 benzene ring.

*D*—H⋯*A*	*D*—H	H⋯*A*	*D*⋯*A*	*D*—H⋯*A*
C20—H20⋯O1^i^	0.95	2.48	3.283 (2)	142
C21—H21⋯O2^ii^	0.95	2.37	3.213 (2)	148
C10—H10*B*⋯*Cg*1^iii^	0.99	2.86	3.624 (2)	135
C15—H15*C*⋯*Cg*1^iv^	0.98	2.93	3.510 (2)	119

Symmetry codes: (i) 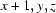; (ii) 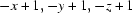; (iii) 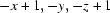; (iv) 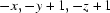.

**Fig. 1.**
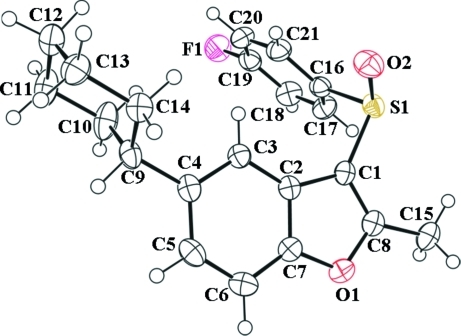
The molecular structure of the title compound with the atom numbering scheme. Displacement ellipsoids are drawn at the 50% probability level. H atoms are presented as a small spheres of arbitrary radius.

**Fig. 2.**
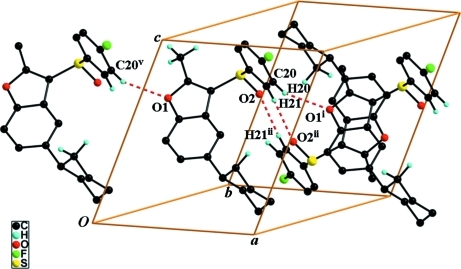
A view of the C—H···O hydrogen bonds (dotted lines) in the crystal structure of the title compound. [Symmetry codes: (i) x + 1, y, z ; (ii) - x + 1, - y + 1, - z + 1; (v) x - 1, y, z ]

**Fig. 3.**
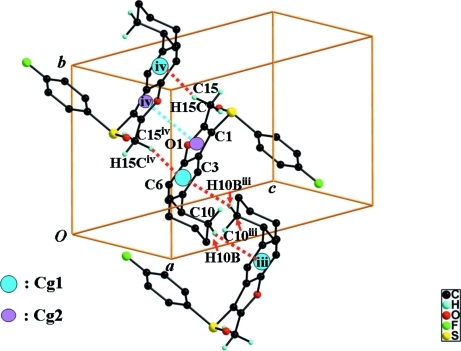
A view of the C—H···π and π–π interactions (dotted lines) in the crystal structure of the title compound. [Symmetry codes: (iii) - x + 1, - y, - z + 1; (iv) - x, - y + 1, - z + 1.]

## supplementary crystallographic information

### Comment

Many compounds having a benzofuran skeleton have attracted much attention owing to their interesting pharmacological properties such as antifungal, antitumor and antiviral, antimicrobial activities (Aslam *et al.*, 2006, Galal *et al.*, 2009, Khan *et al.*, 2005). These compounds widely occur in nature (Akgul & Anil, 2003; Soekamto *et al.*, 2003). As a part of our study of the substituent effect on the solid state structures of 3-(4-fluorophenylsulfinyl)-2-methyl-1-benzofuran analogues (Choi *et al.*, 2010**a*,*b*,c*), we report here on the crystal structure of the title compound.

In the title molecule (Fig. 1), the benzofuran unit is essentially planar, with a mean deviation of 0.004 (1) Å from the least-squares plane defined by the nine constituent atoms. The cyclohexyl ring is in the chair form. The dihedral angle formed by the mean plane of the benzofuran ring and the 4-fluorophenyl ring is 83.55 (4)°.  The molecular packing (Fig. 2) is stabilized by weak intermolecular C—H···O hydrogen bonds; the first one between a 4-fluorophenyl H atom and the furan O atom (Table 1; C20—H20···O1^i^), and the second one between a 4-fluorophenyl H atom and the oxygen of the S═O unit (Table 1; C21-H21···O2^ii^). The molecular packing (Fig. 3) is further stabilized by intermolecular C—H···π interactions; the first one between a cyclohexyl H atom and the benzene ring (Table 1; C10—H10B···Cg1^iii^), and the second one between a methyl H atom and the benzene ring (Table 1; C15—H15C···Cg1^iv^, Cg1 is the centroid of the C2–C7 benzene ring). The molecular packing (Fig. 3) also exhibits aromatic π–π interactions between the furan rings of neighbouring molecules, with a Cg2···Cg2^iv^ distance of 3.541 (2) ° (Cg2 is the centroid of the C1/C2/C7/O1/C8 furan ring).

### Experimental

77% 3-chloroperoxybenzoic acid (224 mg, 1.0 mmol) was added in small portions to a stirred solution of 5-cyclohexyl-3-(4-fluorophenylsulfanyl)-2-methyl-1-benzofuran (306 mg, 0.9 mmol) in dichloromethane (40 mL) at 273 K. After being stirred at room temperature for 4h, the mixture was washed with saturated sodium bicarbonate solution and the organic layer was separated, dried over magnesium sulfate, filtered and concentrated at reduced pressure. The residue was purified by column chromatography (hexane–ethyl acetate, 2:1 v/v) to afford the title compound as a colorless solid [yield 74%, m.p. 422–423 K; *R*_f_ = 0.59 (hexane–ethyl acetate, 2:1 v/v)]. Single crystals suitable for X-ray diffraction were prepared by slow evaporation of a solution of the title compound in ethyl acetate at room temperature.

### Refinement

All H atoms were positioned geometrically and refined using a riding model, with C–H = 0.95 Å  for aryl, 1.00 Å for methine, 0.99 Å for methylene and 0.98 Å for methyl H atoms, respectively. *U*_iso_(H) =1.2*U*_eq_(C) for aryl, methine and methylene, and 1.5*U*_eq_(C) for methyl H atoms.

### Crystal data

**Table:**

C_21_H_21_FO_2_S	*Z* = 2
*M**_r_* = 356.44	*F*(000) = 376
Triclinic, *P*1	*D*_x_ = 1.327 Mg m^−^^3^
Hall symbol: -P 1	Mo *K*α radiation, λ = 0.71073 Å
*a* = 9.2531 (2) Å	Cell parameters from 6765 reflections
*b* = 10.1934 (2) Å	θ = 2.6–27.3°
*c* = 10.8151 (2) Å	µ = 0.20 mm^−^^1^
α = 81.127 (1)°	*T* = 173 K
β = 66.716 (1)°	Block, colourless
γ = 72.248 (1)°	0.29 × 0.23 × 0.18 mm
*V* = 891.73 (3) Å^3^	

### Data collection

**Table:**

Bruker SMART APEXII CCD diffractometer	4073 independent reflections
Radiation source: rotating anode	3436 reflections with *I* > 2σ(*I*)
graphite multilayer	*R*_int_ = 0.027
Detector resolution: 10.0 pixels mm^-1^	θ_max_ = 27.5°, θ_min_ = 2.1°
φ and ω scans	*h* = −11→11
Absorption correction: multi-scan (*SADABS*; Bruker, 2009)	*k* = −13→12
*T*_min_ = 0.943, *T*_max_ = 0.964	*l* = −14→14
15790 measured reflections	

### Refinement

**Table:**

Refinement on *F*^2^	Primary atom site location: structure-invariant direct methods
Least-squares matrix: full	Secondary atom site location: difference Fourier map
*R*[*F*^2^ > 2σ(*F*^2^)] = 0.040	Hydrogen site location: difference Fourier map
*wR*(*F*^2^) = 0.111	H-atom parameters constrained
*S* = 1.04	*w* = 1/[σ^2^(*F*_o_^2^) + (0.0539*P*)^2^ + 0.3464*P*] where *P* = (*F*_o_^2^ + 2*F*_c_^2^)/3
4073 reflections	(Δ/σ)_max_ = 0.001
227 parameters	Δρ_max_ = 0.60 e Å^−^^3^
0 restraints	Δρ_min_ = −0.27 e Å^−^^3^

### Special details

**Table:**

Geometry. All esds (except the esd in the dihedral angle between two l.s. planes) are estimated using the full covariance matrix. The cell esds are taken into account individually in the estimation of esds in distances, angles and torsion angles; correlations between esds in cell parameters are only used when they are defined by crystal symmetry. An approximate (isotropic) treatment of cell esds is used for estimating esds involving l.s. planes.
Refinement. Refinement of F^2^ against ALL reflections. The weighted R-factor wR and goodness of fit S are based on F^2^, conventional R-factors R are based on F, with F set to zero for negative F^2^. The threshold expression of F^2^ > 2sigma(F^2^) is used only for calculating R-factors(gt) etc. and is not relevant to the choice of reflections for refinement. R-factors based on F^2^ are statistically about twice as large as those based on F, and R- factors based on ALL data will be even larger.

### Fractional atomic coordinates and isotropic or equivalent isotropic displacement parameters (Å^2^)

**Table:**

	*x*	*y*	*z*	*U*_iso_*/*U*_eq_	
S1	0.16989 (5)	0.52226 (4)	0.70879 (4)	0.03038 (12)	
F1	0.60813 (14)	0.09572 (12)	0.92500 (12)	0.0523 (3)	
O1	−0.06925 (13)	0.32462 (11)	0.60921 (11)	0.0329 (3)	
O2	0.27322 (16)	0.59605 (12)	0.59571 (12)	0.0426 (3)	
C1	0.10554 (18)	0.41527 (15)	0.64093 (14)	0.0273 (3)	
C2	0.19912 (18)	0.32226 (15)	0.53060 (14)	0.0264 (3)	
C3	0.36263 (18)	0.27915 (15)	0.44540 (14)	0.0280 (3)	
H3	0.4427	0.3139	0.4534	0.034*	
C4	0.40637 (19)	0.18409 (15)	0.34828 (15)	0.0298 (3)	
C5	0.2851 (2)	0.13569 (17)	0.33787 (16)	0.0355 (4)	
H5	0.3163	0.0712	0.2711	0.043*	
C6	0.1226 (2)	0.17765 (18)	0.42034 (16)	0.0361 (4)	
H6	0.0417	0.1445	0.4118	0.043*	
C7	0.08481 (18)	0.27058 (16)	0.51576 (15)	0.0293 (3)	
C8	−0.05259 (18)	0.41298 (16)	0.68288 (15)	0.0299 (3)	
C9	0.5826 (2)	0.13384 (16)	0.25508 (15)	0.0321 (3)	
H9	0.5877	0.0668	0.1940	0.038*	
C10	0.6938 (2)	0.05807 (19)	0.33242 (18)	0.0459 (5)	
H10A	0.6882	0.1212	0.3961	0.055*	
H10B	0.6547	−0.0210	0.3854	0.055*	
C11	0.8704 (2)	0.00624 (19)	0.23672 (18)	0.0466 (5)	
H11A	0.9398	−0.0392	0.2896	0.056*	
H11B	0.8775	−0.0630	0.1781	0.056*	
C12	0.9333 (2)	0.12360 (18)	0.15011 (18)	0.0395 (4)	
H12A	1.0456	0.0863	0.0854	0.047*	
H12B	0.9376	0.1875	0.2080	0.047*	
C13	0.8246 (2)	0.20204 (18)	0.07324 (16)	0.0374 (4)	
H13A	0.8325	0.1414	0.0063	0.045*	
H13B	0.8637	0.2823	0.0238	0.045*	
C14	0.64620 (19)	0.25206 (16)	0.16682 (15)	0.0323 (3)	
H14A	0.5785	0.2953	0.1122	0.039*	
H14B	0.6358	0.3229	0.2252	0.039*	
C15	−0.20513 (19)	0.48483 (18)	0.78899 (17)	0.0375 (4)	
H15A	−0.1830	0.5534	0.8284	0.056*	
H15B	−0.2460	0.4176	0.8593	0.056*	
H15C	−0.2874	0.5310	0.7494	0.056*	
C16	0.30487 (18)	0.39021 (16)	0.77290 (15)	0.0285 (3)	
C17	0.2397 (2)	0.32608 (18)	0.89640 (16)	0.0358 (4)	
H17	0.1250	0.3507	0.9451	0.043*	
C18	0.3418 (2)	0.22589 (19)	0.94899 (17)	0.0407 (4)	
H18	0.2994	0.1806	1.0337	0.049*	
C19	0.5066 (2)	0.19413 (17)	0.87463 (18)	0.0364 (4)	
C20	0.5741 (2)	0.25692 (19)	0.75227 (18)	0.0392 (4)	
H20	0.6888	0.2315	0.7036	0.047*	
C21	0.4712 (2)	0.35849 (18)	0.70112 (16)	0.0351 (4)	
H21	0.5145	0.4056	0.6178	0.042*	

### Atomic displacement parameters (Å^2^)

**Table:**

	*U*^11^	*U*^22^	*U*^33^	*U*^12^	*U*^13^	*U*^23^
S1	0.0334 (2)	0.0275 (2)	0.0277 (2)	−0.00806 (15)	−0.00769 (15)	−0.00389 (14)
F1	0.0523 (7)	0.0492 (6)	0.0629 (7)	−0.0050 (5)	−0.0358 (6)	−0.0011 (5)
O1	0.0274 (5)	0.0389 (6)	0.0319 (6)	−0.0104 (5)	−0.0102 (5)	0.0006 (5)
O2	0.0499 (7)	0.0381 (7)	0.0400 (7)	−0.0193 (6)	−0.0135 (6)	0.0048 (5)
C1	0.0260 (7)	0.0268 (7)	0.0251 (7)	−0.0044 (6)	−0.0074 (6)	−0.0011 (6)
C2	0.0294 (7)	0.0244 (7)	0.0235 (7)	−0.0064 (6)	−0.0093 (6)	0.0012 (5)
C3	0.0289 (7)	0.0280 (7)	0.0258 (7)	−0.0079 (6)	−0.0088 (6)	−0.0006 (6)
C4	0.0351 (8)	0.0260 (7)	0.0236 (7)	−0.0072 (6)	−0.0072 (6)	0.0004 (6)
C5	0.0473 (9)	0.0323 (8)	0.0286 (8)	−0.0125 (7)	−0.0132 (7)	−0.0044 (6)
C6	0.0405 (9)	0.0399 (9)	0.0351 (8)	−0.0173 (7)	−0.0170 (7)	−0.0008 (7)
C7	0.0286 (7)	0.0317 (8)	0.0265 (7)	−0.0081 (6)	−0.0098 (6)	0.0008 (6)
C8	0.0297 (8)	0.0296 (8)	0.0265 (7)	−0.0050 (6)	−0.0098 (6)	0.0022 (6)
C9	0.0369 (8)	0.0276 (8)	0.0250 (7)	−0.0063 (6)	−0.0047 (6)	−0.0054 (6)
C10	0.0420 (10)	0.0397 (9)	0.0323 (8)	0.0052 (8)	−0.0037 (7)	0.0052 (7)
C11	0.0400 (10)	0.0386 (9)	0.0391 (9)	0.0067 (8)	−0.0053 (8)	0.0003 (8)
C12	0.0348 (9)	0.0389 (9)	0.0386 (9)	−0.0017 (7)	−0.0113 (7)	−0.0068 (7)
C13	0.0350 (9)	0.0406 (9)	0.0314 (8)	−0.0106 (7)	−0.0082 (7)	0.0028 (7)
C14	0.0340 (8)	0.0319 (8)	0.0281 (7)	−0.0071 (6)	−0.0106 (6)	0.0006 (6)
C15	0.0272 (8)	0.0412 (9)	0.0345 (8)	−0.0037 (7)	−0.0064 (7)	0.0003 (7)
C16	0.0297 (7)	0.0307 (8)	0.0261 (7)	−0.0107 (6)	−0.0081 (6)	−0.0050 (6)
C17	0.0308 (8)	0.0447 (9)	0.0283 (8)	−0.0121 (7)	−0.0060 (6)	−0.0007 (7)
C18	0.0434 (10)	0.0471 (10)	0.0320 (8)	−0.0160 (8)	−0.0139 (7)	0.0048 (7)
C19	0.0393 (9)	0.0334 (8)	0.0443 (9)	−0.0081 (7)	−0.0233 (8)	−0.0051 (7)
C20	0.0272 (8)	0.0452 (10)	0.0456 (10)	−0.0117 (7)	−0.0095 (7)	−0.0096 (8)
C21	0.0325 (8)	0.0400 (9)	0.0315 (8)	−0.0159 (7)	−0.0056 (7)	−0.0022 (7)

### Geometric parameters (Å, °)

**Table:**

S1—O2	1.4852 (12)	C11—C12	1.515 (3)
S1—C1	1.7524 (16)	C11—H11A	0.9900
S1—C16	1.7956 (16)	C11—H11B	0.9900
F1—C19	1.3588 (19)	C12—C13	1.519 (2)
O1—C8	1.3685 (19)	C12—H12A	0.9900
O1—C7	1.3824 (18)	C12—H12B	0.9900
C1—C8	1.356 (2)	C13—C14	1.528 (2)
C1—C2	1.450 (2)	C13—H13A	0.9900
C2—C7	1.386 (2)	C13—H13B	0.9900
C2—C3	1.396 (2)	C14—H14A	0.9900
C3—C4	1.395 (2)	C14—H14B	0.9900
C3—H3	0.9500	C15—H15A	0.9800
C4—C5	1.403 (2)	C15—H15B	0.9800
C4—C9	1.513 (2)	C15—H15C	0.9800
C5—C6	1.380 (2)	C16—C21	1.380 (2)
C5—H5	0.9500	C16—C17	1.382 (2)
C6—C7	1.378 (2)	C17—C18	1.385 (2)
C6—H6	0.9500	C17—H17	0.9500
C8—C15	1.481 (2)	C18—C19	1.373 (2)
C9—C14	1.531 (2)	C18—H18	0.9500
C9—C10	1.533 (2)	C19—C20	1.371 (2)
C9—H9	1.0000	C20—C21	1.387 (2)
C10—C11	1.525 (2)	C20—H20	0.9500
C10—H10A	0.9900	C21—H21	0.9500
C10—H10B	0.9900		
			
O2—S1—C1	108.26 (7)	C10—C11—H11B	109.4
O2—S1—C16	106.99 (7)	H11A—C11—H11B	108.0
C1—S1—C16	98.14 (7)	C11—C12—C13	111.33 (15)
C8—O1—C7	106.48 (11)	C11—C12—H12A	109.4
C8—C1—C2	107.33 (14)	C13—C12—H12A	109.4
C8—C1—S1	123.11 (12)	C11—C12—H12B	109.4
C2—C1—S1	129.47 (11)	C13—C12—H12B	109.4
C7—C2—C3	119.30 (14)	H12A—C12—H12B	108.0
C7—C2—C1	104.57 (13)	C12—C13—C14	111.94 (13)
C3—C2—C1	136.13 (14)	C12—C13—H13A	109.2
C4—C3—C2	118.81 (14)	C14—C13—H13A	109.2
C4—C3—H3	120.6	C12—C13—H13B	109.2
C2—C3—H3	120.6	C14—C13—H13B	109.2
C3—C4—C5	119.29 (14)	H13A—C13—H13B	107.9
C3—C4—C9	120.33 (14)	C13—C14—C9	111.75 (13)
C5—C4—C9	120.38 (14)	C13—C14—H14A	109.3
C6—C5—C4	122.88 (15)	C9—C14—H14A	109.3
C6—C5—H5	118.6	C13—C14—H14B	109.3
C4—C5—H5	118.6	C9—C14—H14B	109.3
C7—C6—C5	116.01 (15)	H14A—C14—H14B	107.9
C7—C6—H6	122.0	C8—C15—H15A	109.5
C5—C6—H6	122.0	C8—C15—H15B	109.5
C6—C7—O1	125.54 (14)	H15A—C15—H15B	109.5
C6—C7—C2	123.71 (15)	C8—C15—H15C	109.5
O1—C7—C2	110.75 (13)	H15A—C15—H15C	109.5
C1—C8—O1	110.86 (13)	H15B—C15—H15C	109.5
C1—C8—C15	133.48 (16)	C21—C16—C17	121.34 (15)
O1—C8—C15	115.66 (14)	C21—C16—S1	119.74 (12)
C4—C9—C14	111.53 (13)	C17—C16—S1	118.87 (12)
C4—C9—C10	112.00 (13)	C16—C17—C18	119.85 (15)
C14—C9—C10	110.18 (14)	C16—C17—H17	120.1
C4—C9—H9	107.6	C18—C17—H17	120.1
C14—C9—H9	107.6	C19—C18—C17	117.80 (15)
C10—C9—H9	107.6	C19—C18—H18	121.1
C11—C10—C9	111.25 (14)	C17—C18—H18	121.1
C11—C10—H10A	109.4	F1—C19—C20	118.26 (15)
C9—C10—H10A	109.4	F1—C19—C18	118.36 (16)
C11—C10—H10B	109.4	C20—C19—C18	123.37 (16)
C9—C10—H10B	109.4	C19—C20—C21	118.46 (15)
H10A—C10—H10B	108.0	C19—C20—H20	120.8
C12—C11—C10	111.13 (14)	C21—C20—H20	120.8
C12—C11—H11A	109.4	C16—C21—C20	119.14 (15)
C10—C11—H11A	109.4	C16—C21—H21	120.4
C12—C11—H11B	109.4	C20—C21—H21	120.4
			
O2—S1—C1—C8	−130.14 (13)	C7—O1—C8—C15	179.16 (12)
C16—S1—C1—C8	118.88 (13)	C3—C4—C9—C14	−62.29 (18)
O2—S1—C1—C2	46.04 (15)	C5—C4—C9—C14	117.66 (16)
C16—S1—C1—C2	−64.95 (14)	C3—C4—C9—C10	61.72 (19)
C8—C1—C2—C7	−0.05 (16)	C5—C4—C9—C10	−118.33 (17)
S1—C1—C2—C7	−176.70 (12)	C4—C9—C10—C11	179.11 (15)
C8—C1—C2—C3	179.74 (16)	C14—C9—C10—C11	−56.1 (2)
S1—C1—C2—C3	3.1 (3)	C9—C10—C11—C12	57.1 (2)
C7—C2—C3—C4	−0.5 (2)	C10—C11—C12—C13	−55.7 (2)
C1—C2—C3—C4	179.69 (15)	C11—C12—C13—C14	54.27 (19)
C2—C3—C4—C5	0.7 (2)	C12—C13—C14—C9	−54.08 (19)
C2—C3—C4—C9	−179.37 (13)	C4—C9—C14—C13	179.57 (13)
C3—C4—C5—C6	−0.2 (2)	C10—C9—C14—C13	54.54 (18)
C9—C4—C5—C6	179.83 (15)	O2—S1—C16—C21	−11.66 (15)
C4—C5—C6—C7	−0.4 (2)	C1—S1—C16—C21	100.35 (14)
C5—C6—C7—O1	−179.21 (14)	O2—S1—C16—C17	165.84 (13)
C5—C6—C7—C2	0.5 (2)	C1—S1—C16—C17	−82.15 (14)
C8—O1—C7—C6	−179.40 (15)	C21—C16—C17—C18	−1.3 (3)
C8—O1—C7—C2	0.81 (16)	S1—C16—C17—C18	−178.73 (13)
C3—C2—C7—C6	−0.1 (2)	C16—C17—C18—C19	0.0 (3)
C1—C2—C7—C6	179.74 (14)	C17—C18—C19—F1	−179.83 (15)
C3—C2—C7—O1	179.70 (12)	C17—C18—C19—C20	0.4 (3)
C1—C2—C7—O1	−0.47 (16)	F1—C19—C20—C21	−179.32 (15)
C2—C1—C8—O1	0.56 (17)	C18—C19—C20—C21	0.5 (3)
S1—C1—C8—O1	177.47 (10)	C17—C16—C21—C20	2.1 (2)
C2—C1—C8—C15	−179.45 (15)	S1—C16—C21—C20	179.56 (12)
S1—C1—C8—C15	−2.5 (3)	C19—C20—C21—C16	−1.7 (2)
C7—O1—C8—C1	−0.84 (16)		

### Hydrogen-bond geometry (Å, °)

**Table:**

Cg1 is the centroid of the C2–C7 benzene ring.

**Table:**

*D*—H···*A*	*D*—H	H···*A*	*D*···*A*	*D*—H···*A*
C20—H20···O1^i^	0.95	2.48	3.283 (2)	142
C21—H21···O2^ii^	0.95	2.37	3.213 (2)	148
C10—H10B···Cg1^iii^	0.99	2.86	3.624 (2)	135
C15—H15C···Cg1^iv^	0.98	2.93	3.510 (2)	119

Symmetry codes: (i) *x*+1, *y*, *z*; (ii) −*x*+1, −*y*+1, −*z*+1; (iii) −*x*+1, −*y*, −*z*+1; (iv) −*x*, −*y*+1, −*z*+1.
